# Correction: A highly selective ratiometric fluorescent probe for the cascade detection of Zn^2+^ and H_2_PO_4_^−^ and its application in living cell imaging

**DOI:** 10.1039/c8ra90066c

**Published:** 2018-08-06

**Authors:** Kui Du, Shizhen Niu, Li Qiao, Yandong Dou, Qing Zhu, Xinzhi Chen, Pengfei Zhang

**Affiliations:** Key Laboratory of Biomass Chemical Engineering of Ministry of Education, College of Chemical and Biological Engineering, Zhejiang University Hangzhou 310027 P. R. China 15268560133@163.com; College of Material Chemistry and Chemical Engineering, Hangzhou Normal University Hangzhou 310036 P. R. China; Key Laboratory of Bioorganic Synthesis of Zhejiang Province, College of Biotechnology and Bioengineering, Zhejiang University of Technology Hangzhou 310014 China

## Abstract

Correction for ‘A highly selective ratiometric fluorescent probe for the cascade detection of Zn^2+^ and H_2_PO_4_^−^ and its application in living cell imaging’ by Kui Du *et al.*, *RSC Adv.*, 2017, **7**, 40615–40620.

Affiliation *c* was incomplete in the original publication; the corrected version is shown below.

The Royal Society of Chemistry apologises for these errors and any consequent inconvenience to authors and readers.

## Supplementary Material

